# Supplementary material for: Empowerment and utilization of HIV testing among partnered women in Zambia: Evidence from the Zambia demographic and health survey 2018
Source: PLOS Glob Public Health. 2024 Dec 12;4(12):e0002791. doi: 10.1371/journal.pgph.0002791 (PMC11637316; doi:10.1371/journal.pgph.0002791)
Supplement: Attachment — Submitted filename: RESPONSE TO REVIEWERS.docx [file pgph.0002791.s002.docx]

27th May 2024

The Editor: PLOS GLOBAL PUBLIC HEALTH

Dear Editor,

On behalf of my co-authors, we thank you for your valuable comments and the opportunity to submit a revised draft of our manuscript titled “**Women Empowerment and Utilization of HIV Testing among Couples in Zambia: Evidence from the Zambia Demographic and Health Survey 2018**” for consideration of publication in PLOS Global Public Health.

We are thankful to the reviewer for the thorough review, and we believe that the manuscript has been significantly improved with the input.

Sincerely,

Please find our responses in red, highlighting how the manuscript has been modified in response to the reviewer's comments. Line numbers indicate the line numbers were changes have been made in the revised manuscript with Track Changes.

**Editors comment**

The reviewer suggested that you provide more information on the theory of empowerment beyond the index used and explain the limitations of this index with regard to their results and the context of the study.

We have addressed the reviewer's comments by providing a more detailed discussion on the theory of empowerment beyond the index used. Additionally, we have elaborated on the limitations of the index in relation the context of the study. Details of the revisions made are described in relation to each review comment below.

Reviewer # 1

**Introduction**

Line 76 : “Empowerment has been variably defined, with limited data for 77 comparisons across countries, especially in Sub-Saharan Africa”: based on all the evidence you described, what is your definition of womens’ empowerment in this study ?

We have revised the manuscript and included the definition of women’s empowerment in the context of our study. This revision can be found in lines 78-83 of the revised manuscript with track changes.

**Methodology**

1. Line 123: “the country recorded a drop of 43.7% in HIV testing in 2020 compared to 2019”: are there differences between men and women?

Thank you so much for noticing this gap in information. We have revised the manuscript by adding information to the statement to clarify that the recorded drop in HIV testing in 2020 compared to 2019 was similar for both males and females. This revision can be found in lines 120-131 of the revised manuscript with track changes

2. Line 168: “Our study was restricted to married/partnered Women with a focus on the couples recode section of the data since the SWPER index considers only married or partnered women”.: That's a very important point. You could also discuss the restriction of the empowerment index to women who are married or in a couple: is the empowerment of women essentially linked to the fact of being married or having a partner? What about women who are not married or do not have a partner?

Thank you for your insightful comment. We have acknowledged this as a limitation to our study, which focused on married or partnered women due to the nature of the SWPER index. We believe that empowerment is not necessarily linked to marital status and hence this restriction raises important questions about the relationship between empowerment and marital status. Many empowered women may not be married or may plan to marry later in life. Moreover, disabled women and sex workers, who are among the most marginalized and disempowered groups, may be less inclined to be married, thus rendering them ineligible for inclusion in the study. Similarly, our findings cannot be generalized to adolescents, many of whom are unmarried. We have recognized the potential implications of this restriction in our limitations and the need for further research that explores the empowerment of both partnered and unpartnered women. This revision can be found in lines 547-553 of the revised manuscript.

3. Table 2 and reference 42 : the link to your reference does not work to see the details of the equations used to calculate SWPER

Thank you so much for noticing this error in the citation. We have revised the reference to the link leading to details of equations used to calculate the SWPER. This can be found in line 213.

4. Tables 1 and 2 : mention the sources above or below the tables

We have mentioned the sources of information for Tables 1 & 2 above each table

**Results**

1. Table 3: Since pregnant women are tested for HIV, perhaps you could carry out a sensitivity test, taking into account women who have not given birth in the last five years or who have never given birth? It could be interesting to see if the influence of women's empowerment on access to HIV testing is independent of the measures put in place for those who are pregnant. For example, will a woman in a partnered relationship go for a screening test on her own if she is not pregnant or does not have children or has not had children in the last 5 years ? My comment is also echoing to the following sentences in the discussion (line 410-415): “The diminished significance of associations with HIV testing after adjusting for the effects of other variables may be attributed to a potential confounding factor. It is plausible that the higher proportion of women in our study who had given birth in the last 5 years could have influenced the results. In Zambia, HIV testing is done routinely during pregnancy as part of services offered during antenatal care attendance, and this might have played a role in shaping women’s decision to test for HIV”.

Thank you so much for this insightful comment. To understand if the influence of women’s empowerment on HIV testing was independent of the measures in place to test for HIV in all women who attend antenatal care, we have conducted separate logistic regression analyses among the two distinct groups of women: those who gave birth in the last five years and those who did not (models 7 and 8) respectively. Our results revealed that decision-making was the only domain of women's empowerment that remained significant in both categories, consistent with the findings from the main sample. This suggests that decision-making autonomy plays a crucial role in influencing HIV testing behavior, regardless of measures put in place to test all women during antenatal care attendance. This addition can be found in Table 9 and elaborated in lines 400-422 of the revised manuscript. Furthermore, we have added to the discussion results of this analysis in lines 476-498 of the revised manuscript.

2. Line 275: “(Table 4) presents the results of the associations”: delete the parenthesis

We have deleted the parenthesis.

**Discussion**

1. Line 419: “The observed differences in findings may stem from variations in study populations, contexts, and methodological approaches used in these studies.” Previous studies on empowerment theory have indicated that empowerment is a reality dependent on context and culture. You could therefore go further and discuss this point in your results.

Thank you for highlighting this important aspect. We have expanded our discussion on the influence of context and culture on women's empowerment in lines 466-475.

2. Line 428: Aren't there other possible reasons: the age at which it is possible to have a screening test without parental consent? Sexuality education for young people? easier access to screening for young people without children?

Thank you for your insightful comment. We have addressed these potential reasons in our revised manuscript. We have discussed the age at which individuals can have a screening test without parental consent in Zambia, and taken this into consideration in our interpretation of the observed trends in HIV testing behavior among different age groups. This revision can be found in lines 515-521 of the revised manuscript

**Other Comments**

1. Perhaps you could include an appendix describing the characteristics of women according to their level of empowerment. This would give a more detailed idea of the women concerned by each situation and could also help you to comment more effectively on the effect of other characteristics by making the link with empowerment.

Thank you for this valuable comment. We have included a table (Table 6) describing women empowerment domains by various background characteristics. Additionally, we have conducted chi-square tests to determine the significance of the observed associations between these characteristics and the empowerment domains. This can be found in Table 6 and lines 320-326 in the revised manuscript.

2. You might consider adding a grid adapted to your study, based on the recommendations for reporting quantitative studies: in the absence of a guideline for analysing secondary data, it seems to me that the STROBE guide could be useful to you: <https://www.equator-network.org/reporting-guidelines/coreq/>.

Thank you so much for this insight. We have followed the checklist adapted to our study for reporting quantitative cross-sectional studies based on the STROBE guidelines and added the resultant grid to our supplemental materials.
