# Supplementary material for: Empowerment and utilization of HIV testing among partnered women in Zambia: Evidence from the Zambia demographic and health survey 2018
Source: PLOS Glob Public Health. 2024 Dec 12;4(12):e0002791. doi: 10.1371/journal.pgph.0002791 (PMC11637316; doi:10.1371/journal.pgph.0002791)
Supplement: Attachment — Submitted filename: Abstrac2.docx [file pgph.0002791.s003.docx]

Abstract

- This study examined associations between women's empowerment and HIV testing among married and partnered women. – suggest rewording this too: This study examined associations between empowerment and HIV testing among married and partnered women.
- lowly empowered – less empowered?

Introduction

Overall, there are many places in the introduction that require references, I have listed some in the feedback below.

- Women’s access to HIV prevention services, such as HIV testing depends significantly on the level of empowerment. – this requires a reference, but also to make it clear that women’s ability to test is mediated by empowerment not utterly dependent on it - there are many other reasons why a woman may not test besides empowerment.
- Women who are empowered can make independent decisions regarding their health, including HIV testing without depending on their husbands or partners – reference
- This has resulted in a disproportion in HIV transmission 54 between boys and girls, with girls and young women carrying the heaviest burden of HIV prevalence (13%) compared to boys and men (7%) – again, there are other reasons why girls have heightened vulnerability including biological mechanisms – therefore I would say “This has contributed to a disproportionation…”
- The extent to which a woman has been empowered significantly influences her accessibility to HIV testing – reference
- study therefore assessed the effects of women's empowerment on HIV testing among couples – I do not think that this is an accurate description of what the study has done. “among couples” implies that the study has looked at indicators for both partners in dyad – whereas this study looks at data only from the female partner. It would be more accurate to say HIV testing among women in relationships.
- It would also be helpful to have some literature on couples testing and what work has been done in this area - plus the benefits of couples making decisions together as well.

Material and methods

- The Republic of Zambia is in Southern Africa and is one of the low-income countries – rewrite to: The Republic of Zambia is in Southern Africa and is a low-income country.
- “…possessing biased gender social norms, with…” should this read unequal gender norms?
- “This has resulted in gender inequities in health with resultant higher HIV infection among females compared to males in the country” - this implies causation - it is one of the factors contributing to increased risk of HIV infection- not the sole cause.
- Moreover, the country recorded a drop of 126 43.7% in HIV testing in 2020 compared to 2019, with similar decreases observed across both sexes [39]. – considering how this is placed in the paragraph, this again implies causation, and that this is a result of gender norms when there may be multiple reasons.

Dependant variable

- If the dependant variable is “ever tested for HIV” - how is this contemporaneous with current empowerment (and it effect on testing), and how can this be connected to the relationship they are in now? The HIV test could have happened at any point in their lifetime?

Results

- How was wealth defined and measured?
- How was age accounted for as a potentially confounding factor – i.e. older women would have had more opportunity to test by virtue of being older, also more likely to have had children and been to ANC services – this may confound any relationships?
- In the results section, the authors switch between significantly varied, and significant associations – please consistently use associations to describe the significant findings of the Chi square.
- The numbers (n/N) are missing from table 6.
- Were there any indicators of relationship quality, partner characteristics in the DHS survey or length of relationship? These would be important indicators considering the focus of the paper.
- In the recent birth sub-analysis, is there potential correlations and confounding to adding wealth and education to the model considering by virtue older age, women are more likely to have accumulated more wealth and have completed school – 20-year-old women may still be finishing school?
- In the sub-analysis with women that gave birth in the last 5 years - the women in the no group may have still given birth before - and may have still tested before - which is problematic because the dependent variable is ever tested. why not compare it to the group of women who reported that they have never tested before?

Discussion

- It is presumed that an empowered woman, whether through cultural, political, or professional means possesses the confidence to independently choose to undergo HIV testing [15]. This autonomy ensures that she is not reliant on her husband or partner to make decisions regarding whether to undergo HIV testing – what about the importance of relational quality, and co-decision making in couples? It is possible that women who have tested also have better relationships that women who have not – but this has not been measured. So, while empowerment is obviously critical, the quality of relationships is also important.
- Women with some form of education are more likely to be aware of the importance of HIV testing – reference
- I think that the title of the article and some of the wording should better reflect that this is an analysis of data from married women or women in relationships – and not couples. Couples implies that the analysis considered data from both partners which this analysis does not.
- It would also be useful to have more reflection on what these results mean for intervention efforts beyond the superficial – “empowerment programmes are critical” How do we take these findings and apply them in a meaningful way?
