# Supplementary material for: Empowerment and utilization of HIV testing among partnered women in Zambia: Evidence from the Zambia demographic and health survey 2018
Source: PLOS Glob Public Health. 2024 Dec 12;4(12):e0002791. doi: 10.1371/journal.pgph.0002791 (PMC11637316; doi:10.1371/journal.pgph.0002791)
Supplement: Attachment — Submitted filename: RESPONSE TO REVIEWERS.docx [file pgph.0002791.s004.docx]

4th October 2024

The Editor: PLOS GLOBAL PUBLIC HEALTH

Dear Editor,

On behalf of my co-authors, we thank you for your valuable comments and the opportunity to submit a revised draft of our manuscript titled “**Women Empowerment and Utilization of HIV Testing among Couples in Zambia: Evidence from the Zambia Demographic and Health Survey 2018**” for consideration of publication in PLOS Global Public Health.

We are thankful to the second reviewer for their thorough review, and we believe that the manuscript has been significantly improved with the input.

Sincerely,

Please find our responses in red, highlighting how the manuscript has been modified in response to the reviewer's comments. Line numbers indicate the line numbers were changes have been made in the revised manuscript with Track Changes.

**Reviewer # 1**

1. I would like to thank the authors for their responses to my comments. Just 2 comments: Line 229: “We constructed six logistic regression models”: the authors should correct, they now have 8 models, if I am not mistaken.

Thank you for bringing this to our attention. You are correct; we have updated the number of models to eight and made corrections on line 229 to reflect this

2. Line 270: It seems a bit strange to start a sentence with brackets. It may be done, but if it's possible to start the presentation of tables directly without putting them in brackets, except in the middle or at the end of a sentence, it would be more reader-friendly.

Thank you for your insightful comment. We have revised the sentence and removed the brackets.

**Reviewer # 2**

**Abstract**

1. This study examined associations between women's empowerment and HIV testing among married and partnered women. – suggest rewording this too: This study examined associations between empowerment and HIV testing among married and partnered women.

We have revised the sentence in line 16 and it now reads “This study examined associations between empowerment and HIV testing among married and partnered women”.

2. lowly empowered – less empowered?

Thank you for this insight. We have reworded the sentence in line 23-24 from lowly empowered to less empowered

**Introduction**

1. Overall, there are many places in the introduction that require references, I have listed some in the feedback below.

Thank you so much for this insight. We have revised the introduction by including additional references.

2. Women’s access to HIV prevention services, such as HIV testing depends significantly on the level of empowerment. – this requires a reference, but also to make it clear that women’s ability to test is mediated by empowerment not utterly dependent on it - there are many other reasons why a woman may not test besides empowerment.

Thank you for your valuable feedback. We have revised the sentence by rewording to clarify that women's access to HIV testing is influenced by and not dependent on level of empowerment. Additionally, we have included references to support this point. This can be found in lines 36-37.

3. Women who are empowered can make independent decisions regarding their health, including HIV testing without depending on their husbands or partners – reference

We have added a reference to the statement

4. This has resulted in a disproportion in HIV transmission 54 between boys and girls, with girls and young women carrying the heaviest burden of HIV prevalence (13%) compared to boys and men (7%) – again, there are other reasons why girls have heightened vulnerability including biological mechanisms – therefore I would say “This has contributed to a disproportionation…”

Thank you for your insightful comment! We agree that multiple factors contribute to girls' vulnerability to HIV, including biological factors. We have, therefore, revised the statement to more accurately indicate that there are other factors that come into play

5. The extent to which a woman has been empowered significantly influences her accessibility to HIV testing – reference

We have included references to this

6. study therefore assessed the effects of women's empowerment on HIV testing among couples – I do not think that this is an accurate description of what the study has done. “among couples” implies that the study has looked at indicators for both partners in dyad – whereas this study looks at data only from the female partner. It would be more accurate to say HIV testing among women in relationships.

Thank you for your careful observation. We have revised the sentence to accurately reflect the objective of the study. The revised sentence now reads “This study therefore assessed the effects of empowerment on HIV testing among coupled women in Zambia”. Lines 100-101

6. It would also be helpful to have some literature on couples testing and what work has been done in this area - plus the benefits of couples making decisions together as well.

Thank you for your suggestion. We have incorporated relevant literature on couples testing and the benefits of joint decision-making in lines 68-72

**Material and methods**

1. The Republic of Zambia is in Southern Africa and is one of the low-income countries – rewrite to: The Republic of Zambia is in Southern Africa and is a low-income country.

We have rewritten this sentence

2. “…possessing biased gender social norms, with…” should this read unequal gender norms?

Thank you for this, we revised the sentence.

3. “This has resulted in gender inequities in health with resultant higher HIV infection among females compared to males in the country” - this implies causation - it is one of the factors contributing to increased risk of HIV infection- not the sole cause.

Thank you for this insight. We have revised the sentence to more accurately indicate that there are other factors at play. The revised sentence now reads “This gender inequity in health has contributed to the observed higher HIV infection among females compared to males in the country”

4. Moreover, the country recorded a drop of 126 43.7% in HIV testing in 2020 compared to 2019, with similar decreases observed across both sexes [39]. – considering how this is placed in the paragraph, this again implies causation, and that this is a result of gender norms when there may be multiple reasons.

Thank you for this important observation. We have included a sentence to emphasize that other factors, beyond gender norms, may have contributed to the observed decline in the HIV testing rate.

**Dependent Variable**

1. If the dependant variable is “ever tested for HIV” - how is this contemporaneous with current empowerment (and it effect on testing), and how can this be connected to the relationship they are in now? The HIV test could have happened at any point in their lifetime?

Thank you for your thoughtful comment! We appreciate your concerns regarding the dependent variable of "ever tested for HIV."

In this study, the choice of "ever tested for HIV" as the dependent variable was made to capture a broad understanding of individuals’ engagement with HIV testing throughout their lives. While this variable provided valuable insights into access to and utilization of testing services, we acknowledge that it may not fully reflect the impact of current empowerment on testing behaviors. The variable reflects historical testing behavior rather than current testing practices, making it difficult to establish a direct connection between contemporary levels of women's empowerment and their recent decisions to undergo HIV testing.

We have acknowledged the potential disconnect between historical HIV testing behavior and present empowerment contexts and included this as a limitation of the study in lines 551-554. Thank you for highlighting this important consideration!

**Results**

1. How was wealth defined and measured?

In the DHS survey, wealth was measured using household ownership of selected assets, such as televisions and bicycles; materials used for housing construction; and types of water access and sanitation facilities. By summing household asset questions, respondents are categorized into five groups from poorest to wealthiest. For this study, Wealth index was collapsed into three categories (poor, middle, rich). This is described on page 197.

2. How was age accounted for as a potentially confounding factor – i.e. older women would have had more opportunity to test by virtue of being older, also more likely to have had children and been to ANC services – this may confound any relationships?

Thank you for this question. We accounted for age as a potentially confounding variable in all our analyses. In the adjusted models, our findings indicated that women aged 20 years or more had higher odds of testing for HIV compared to those younger than 20. This aligned with prior studies that reported similar results. These results, together with possible explanations for the observed trend are discussed on pages 486-500 in the discussion section

3. In the results section, the authors switch between significantly varied, and significant associations – please consistently use associations to describe the significant findings of the Chi square.

Thank you for this feedback. We have ensured consistency in our language by using "associations" to describe all significant findings from the Chi-square analyses throughout the section.

4. The numbers (n/N) are missing from table 6.

Thank you for this observation. The numbers in Table 6 are represented in percentages hence we did not include (n/N) in the table. This was done to reduce the bulkiness of the table and enhance readability.

5. Were there any indicators of relationship quality, partner characteristics in the DHS survey or length of relationship? These would be important indicators considering the focus of the paper.

Thank you for your insightful question. The SWPER index used to measure women's empowerment in our analysis does not incorporate indicators of relationship quality. While partner characteristics and the length of relationships can be important indicators, they were not included in the DHS survey data we utilized. Our focus was primarily on the specific dimensions of women's empowerment as outlined in the SWPER index. However, we acknowledge the significance of relationship quality and appreciate your suggestion for future research directions that could explore these aspects more thoroughly.

6. In the recent birth sub-analysis, is there potential correlations and confounding to adding wealth and education to the model considering by virtue older age, women are more likely to have accumulated more wealth and have completed school – 20-year-old women may still be finishing school?

Thank you for your comment. In our analyses, age, wealth, and level of education were indeed found to be significant confounders across all models. We adjusted for these variables in the subanalyses (models 7 and 8) to ensure robust results.

7. In the sub-analysis with women that gave birth in the last 5 years - the women in the no group may have still given birth before - and may have still tested before - which is problematic because the dependent variable is ever tested. why not compare it to the group of women who reported that they have never tested before?

Thank you for your insightful comment. Our primary focus for the sub-analysis was to investigate if the influence of women empowerment on HIV testing was independent of recent childbirth. Across all models (1-6), we found that women who gave birth in the last five years were consistently more likely to have tested for HIV compared to those who did not. To explore this relationship further, we conducted separate logistic regression analyses for the two distinct groups. This approach allowed us to assess whether the impact of women’s empowerment on HIV testing was independent of recent childbirth, rather than childbirth as a whole

**Discussion**

1. It is presumed that an empowered woman, whether through cultural, political, or professional means possesses the confidence to independently choose to undergo HIV testing [15]. This autonomy ensures that she is not reliant on her husband or partner to make decisions regarding whether to undergo HIV testing – what about the importance of relational quality, and co-decision making in couples? It is possible that women who have tested also have better relationships that women who have not – but this has not been measured. So, while empowerment is obviously critical, the quality of relationships is also important.

Thank you for your thoughtful comment. We acknowledge the importance of relationship quality in understanding women’s empowerment and decision-making regarding HIV testing. However, the DHS data does not include indicators for relationship quality, which limits our ability to explore this dimension in our analysis.

We agree that further studies are warranted to incorporate measures of relationship quality and co-decision-making among couples. This would provide a more comprehensive understanding of how these factors interact with women’s empowerment and influence their decisions to undergo HIV testing. Thank you for highlighting this important aspect, and we appreciate your valuable insights!

2. Women with some form of education are more likely to be aware of the importance of HIV testing – reference

We have added a reference to this

3. I think that the title of the article and some of the wording should better reflect that this is an analysis of data from married women or women in relationships – and not couples. Couples implies that the analysis considered data from both partners which this analysis does not.

Thank you for this thoughtful insight. We have edited the title to “Empowerment and Utilization of HIV Testing among Partnered Women in Zambia: Evidence from the Zambia Demographic and Health Survey 2018”. We hope this best reflects the study

4. It would also be useful to have more reflection on what these results mean for intervention efforts beyond the superficial – “empowerment programmes are critical” How do we take these findings and apply them in a meaningful way?

Thank you for this insight. We have included a section “Implications for Policy and Practice” and highlighted targeted intervention efforts that could foster HIV testing among partnered women. Pages 521-537
